# Preservative effects of clove extract against *Alternaria alternata* on postharvest strawberries and associated shifts in microbial communities

**DOI:** 10.3389/fmicb.2026.1736375

**Published:** 2026-02-17

**Authors:** Yang Wu, Mingxue Chang, Yihan Liu, Tong Chen, Zexia Li, Weiwei Li, Dingkun Liu, Li He, Huiming Sun

**Affiliations:** 1 Jinggangshan University Institute of Modern Agricultural Development, Ji’an, China; 2 College of Life Sciences, Jinggangshan University, Ji’an, China

**Keywords:** *Alternaria alternate*, clove extract, microbial community, minimum fungicidal concentration, preservative effect, strawberry

## Abstract

**Introduction:**

Strawberries are highly perishable postharvest due to infection by pathogens such as *Alternaria alternata*, and chemical fungicides have drawbacks like residues and resistance. Clove extract exhibits broad-spectrum antimicrobial activity, but its targeted inhibitory threshold and mechanism on strawberry postharvest pathogens remain unclear.

**Methods:**

The minimum fungicidal concentration (MFC) of clove extract against *A. alternata* was determined. “Benihoppe” strawberries were treated with MFC (30 mg/mL) and stored at 25°C/90% RH. Key quality indicators (firmness, VC content, weight loss rate, etc.) were monitored, and microbial community structure was analyzed via high-throughput sequencing.

**Results:**

The MFC of clove extract against *A. alternata* was 30 mg/mL, which reduced spore survival rate to 40.0% and increased cell membrane permeability. After 8 days of storage, the treatment group showed 68.3% higher firmness, 52.5% higher VC content, and 46.4% lower weight loss rate than the control. The abundance of spoilage microorganisms (e.g., *Alternaria*, Pantoea) decreased, while beneficial bacteria (e.g., *Bacillus*) increased in the treatment group.

**Discussion:**

Clove extract preserves strawberries by directly damaging the cell membrane of *A. alternata* and reshaping the fruit surface microbial community, providing a green alternative for postharvest preservation.

## 1 Introduction

The strawberry (*Fragaria × ananassa*) belongs to the Rosaceae family and is renowned for its sweet and palatable fruit. Currently, it has emerged as a highly economically valuable fruit crop in China. According to the Food and Agriculture Organization of the United Nations (FAO), the strawberry yield in China reached 27,025.8 kg per hectare in 2023, highlighting its status as a high-value economic crop (FAO, 2024). Among cultivated varieties, the “Benihoppe” strawberry (*Fragaria × ananassa* “Benihoppe”) has become a dominant commercial cultivar in China, favored for its delicate texture, balanced sweet-tart flavor profile, and rich nutritional composition including vitamin C and anthocyanins. However, postharvest preservation remains a critical challenge for the industry. With an exceptionally thin epidermis (5–10 μm), a water content exceeding 90%, and a non-climacteric nature accompanied by vigorous postharvest physiological activity, strawberries are particularly susceptible to pathogen infection following mechanical damage. Under ambient storage conditions, decay rates typically range from 30 to 50%, with shelf life generally limited to less than 7 days, significantly constraining distribution ranges and commercial value (Li et al., 2017; Satitmunnaithum et al., 2022).

The main cause of post-harvest decay of strawberries is the infection by pathogenic bacteria. Among them, *A. alternata* is one of the key pathogenic bacteria that have attracted attention in the research on post-harvest diseases of strawberries (Al-Rahbi et al., 2021). *A. alternata* can spread conidia through air and rain, and invade the strawberries through mechanical wounds on the surface of the fruits, natural pores, or even intact epidermis, and is more likely to break through the epidermal barrier in a high-humidity environment (Li et al., 2024). In storage environments with temperatures ranging from 15 to 28°C and high humidity (the optimal relative humidity for the growth and sporulation of *Alternaria alternata* is 95–100%), *A. alternata* can rapidly colonize and infect strawberry fruits. Existing studies have confirmed that this fungus has a wide growth temperature range (approximately 1–35°C), with 20–30°C being the optimal temperature interval for its growth and sporulation. After infecting strawberries, it causes black-brown lesions on the fruits, and as the disease progresses, it further leads to fruit softening and decay. Besides *A. alternata, Botrytis cinerea*, *Penicillium* sp., and spoilage bacteria (such as *Pantoea*) also often work together to form a composite infection, further increasing the risk of decay (Raynaldo et al., 2024). In this study, we focused on *Alternaria alternata*, as the primary target organism.

At present, the preservation of strawberries mainly relies on the combination of low-temperature storage and chemical fungicides. However, low temperature can cause water-stained cold damage spots on the fruits, and chemical agents have problems of residue and resistance, which are contrary to consumers’ demand for “green and residue-free” agricultural products. Under this background, plant-based preservatives, due to their safety and antimicrobial activity, have become an ideal alternative to chemical agents. These extract solutions are rich in active components such as phenols and terpenes, which cannot only inhibit the growth of pathogenic bacteria but also delay the oxidation and aging of fruits. They have shown significant potential in fruit and vegetable preservation (Chen et al., 2023; Bhat and Stamminger, 2015).

*Syzygium aromaticum*, as a plant with both medicinal and edible properties, its extract has been widely verified for its antimicrobial effect. Eugenol, as the core active component of the extract, can bind to the lipid components of the pathogenic bacteria’s cell membrane, damaging the integrity of the membrane structure and causing the leakage of intracellular nucleic acids, proteins, etc.; at the same time, it can inhibit the activities of key metabolic enzymes such as succinate dehydrogenase and chitinase, blocking energy supply and cell wall synthesis. Studies have shown that the *Syzygium aromaticum* extract has significant inhibitory effects on bacteria such as *Escherichia coli* and *Staphylococcus aureus* (Cai and Wu, 1996; Hu et al., 2018), as well as fungi such as *Penicillium* and *Rhizopus*. In the preservation of fruits and vegetables like tomatoes and grapes, it can reduce the invasion of pathogenic bacteria and extend the storage period. Although the antimicrobial potential of the *Syzygium aromaticum* extract has been confirmed, there are still deficiencies in the research on this special berry, strawberry: Firstly, the targeted inhibitory threshold (such as the minimum bactericidal concentration) for post-harvest pathogenic bacteria (including *Alternaria alternata*) of strawberry has not been clearly defined; Secondly, in the actual preservation of strawberries, whether it is possible to reduce spoilage by regulating the microbial community balance and the related mode of actions still lack systematic data. Based on this, this study takes red-fleshed strawberry as the object, first determines the minimum fungicidal concentration (MFC) of the *Syzygium aromaticum* extract targeting *Alternaria alternata*, and then treats the strawberries with the MFC concentration. By monitoring quality indicators (hardness, VC content, etc.) and analyzing the changes in the microbial community, the preservation effect and mode of action are revealed, providing theoretical support for the green preservation of strawberries. Therefore, this study aimed to describe the preservative effect of clove extract on postharvest strawberries by evaluating its impact on (i) the viability of a target spoilage fungus (*A. alternata*), (ii) key fruit quality indicators, and (iii) the composition of the fruit surface microbial community, to explore the potential associations between these factors.

## 2 Materials and methods

### 2.1 Materials and reagents

The *Fragaria × ananassa* “Benihoppe” strawberries were free from damage, pests, and diseases, harvested from the orchard in Ji’an, Jiangxi, and transported to the laboratory within 2 h. *Alternaria*: A strain isolated from decayed strawberries, preserved on Potato Dextrose Agar (PDA) slants at 4°C in our laboratory. Clove extract: Aqueous clove extract was prepared by adding clove powder to distilled water at a ratio of 1:10 (g:mL). The mixture was heated in a water bath at 60°C for 2 h, then filtered. The filtrate was concentrated by rotary evaporation at 60°C to obtain a stock solution of 100 mg/mL (expressed as the concentration of the original plant material). The stock solution was stored at 4°C in the dark for no more than 1 week before use. Working solutions of 0, 15, 20, 25, and 30 mg/mL were prepared fresh by dilution with sterile distilled water on the day of the experiment. Reagents: Potato Dextrose Agar (PDA) medium, Potato Dextrose Broth (PDB) liquid medium (Qingdao Haibo); 3-(4,5-dimethylthiazol-2-yl)-2,5-diphenyltetrazolium bromide (MTT) staining agent (Shanghai Aladdin); 2,6-dichlorophenolindophenol sodium salt (China National Pharmaceutical Group).

### 2.2 Instruments and equipment

UV-2600 UV-Vis Spectrophotometer (Shimadzu); GY-4 Portable Hardness Tester (Zhejiang Top); Illumina MiSeq Sequencing Platform (Shanghai Personal Biotechnology); Constant Temperature Water Bath (Shanghai Lichen); Clean Bench (Suzhou Purification).

### 2.3 Experimental methodology

#### 2.3.1 Cultivation of *Alternaria* and preparation of spore suspension

According to the method of Gao et al. (2016) with slight modifications, *Alternaria* was inoculated onto PDA medium and cultured at 25°C for 7 days; the surface of the medium was rinsed with sterile water, filtered through sterile gauze to obtain a spore suspension, and the concentration was adjusted to 1 × 10^8^ CFU/mL using a hemocytometer.

#### 2.3.2 Determination of MFC of clove extract against *Alternaria alternata*

The minimum fungicidal concentration (MFC) was determined against the vegetative mycelial form of *A. alternata*, as this stage is directly responsible for tissue invasion and decay development in fruits. According to the method of Feng et al. (2023) with slight modifications, Clove extract dilutions (15, 20, 25, 30 mg/mL) were prepared. For each concentration, 1 mL was mixed with 9 mL of molten PDA to prepare drug-incorporated agar plates. After solidification, a 5-mm mycelial disk taken from the actively growing edge of a 7-day-old colony was placed at the center of each plate. Control plates were prepared with 1 mL of sterile distilled water mixed with 9 mL PDA. All plates were incubated at 25°C for 48 h. The MFC was defined as the lowest concentration that resulted in no visible colony growth. Each treatment, including the control, was performed in triplicate (*n* = 3).

#### 2.3.3 Determination of antifungal mode of action

Spore survival rate (MTT staining method): A volume of 100 μL of spore suspension was mixed with100 μL of 30 mg/mL clove extract. The mixture was left to stand at 25°C for 24 h; then centrifuged at 4,000 r/min for 5 min. After discarding the supernatant, 100 μL of MTT staining agent (0.5 mg/mL) was added, and the solution was incubated at 36°C in the dark for 48 h. The blue surviving spores were counted using a hemocytometer, and the survival rate was calculated rate:


$$S\,u\,r\,v\,i\,v\,a\,l\,r\,a\,t\,e=\frac{N\,u\,m\,b\,e\,r\,o\,f\,b\,l\,u\,e\,s\,p\,o\,r\,e\,s}{T\,o\,t\,a\,l\,n\,u\,m\,b\,e\,r\,o\,f\,s\,p\,o\,r\,e\,s}\times 100\%$$

Leakage of nucleic acid and protein: The method of Sun et al. (2023) was followed. Briefly, 0.5 g of fungal mycelia was added to a mixture of 950 μL of sterile water and 50 μL of 30 mg/mL clove extract, and shaken at 25°C. Samples were taken at 1, 2, and 4 h, centrifuged at 8,000 r/min for 10 min, and the OD_260_ (nucleic acid) and OD_280_ (protein) of the supernatant were measured using a UV spectrophotometer.

#### 2.3.4 reservation experiment of the *Fragaria × ananassa* “Benihoppe” strawberries

Sample Treatment: Strawberries were randomly divided into two groups, with 30 fruits in each group, and 3 replicates were performed. Control group (CK group): treated by soaking in sterile distilled water for 5 min; Treatment group (DX group): treated by soaking in 30 mg/mL clove extract for 5 min. After natural air-drying, the strawberries were stored at 25°C with 90% relative humidity, and samples were taken for measurement at 0, 2, 4, 6, and 8 days. Hardness: Hardness was measured using a GY-4 hardness tester on the equatorial surface of the fruit after removing 1 mm of the skin, and the average of 3 repetitions was taken; Vitamin C content: This was determined by the 2,6-dichloroindophenol titration method, with reference to GB 5009.86–2016 “Food Safety National Standard—Determination of Vitamin C in Food.” The result is expressed as mg/100 g fresh weight.

#### 2.3.5 Microbial community analysis

Total genomic DNA was extracted from 40 g of homogenized strawberry fruit surface tissue using the DNeasy PowerSoil Pro Kit (Qiagen, Germany) according to the manufacturer’s instructions. The fungal *ITS1* region was amplified using primers *ITS1F* (5’-CTTGGTCATTTAGAGGAAGTAA-3’) and *ITS2* (5’-GCTGCGTTCTTCATCGATGC-3’). The bacterial *16S rRNA* gene V4-V5 region was amplified using primers 515F (5’-GTGCCAGCMGCCGCGGTAA-3’) and 907R (5’-CCGTCAATTCCTTTGAGTTT-3’). PCR products were purified and paired-end sequenced (2 × 250 bp) on an Illumina MiSeq platform by Personalbio Co., Ltd (Shanghai, China). Raw sequencing reads were processed using QIIME2 (version 2023.9). Sequences were demultiplexed, quality-filtered using the *DADA*2 plugin to remove low-quality reads and chimeras, and clustered into amplicon sequence variants (ASVs). After processing, an average of 50,000 high-quality reads per sample were obtained for downstream analysis. Taxonomic assignment was performed using the *SILVA* 138 database for bacteria and the *UNITE* database for fungi. Microbial community composition at the genus level was analyzed, and differential abundance between groups was identified using random forest analysis. The relationship between microbial community structure and fruit quality indicators was visualized using Redundancy Analysis (RDA).

### 2.4 Data analysis

Data are presented as mean ± standard deviation (SD). Normality and homogeneity of variances were verified using the *Shapiro-Wilk* test and Levene’s test, respectively. For data meeting these assumptions, differences among storage days within the same treatment group were analyzed by one-way analysis of variance (*ANOVA*) followed by Tukey’s *post-hoc* test. Differences between the control (CK) and treatment (DX) groups at each time point were compared using independent samples *t*-test. A significance level of P < 0.05 was applied. All statistical analyses were performed using SPSS 22.0 (IBM Corp., United States). For microbial community data, permutational multivariate analysis of variance (PERMANOVA) based on *Bray-Curtis* distances was performed using QIIME2 to test for overall structural differences between groups.

## 3 Results

### 3.1 The MFC and antifungal effects of clove extract against *Alternaria alternata*

#### 3.1.1 Determination of minimum fungicidal concentration

As shown in Table 1, the clove extract at concentrations of 15∼25 mg/mL exhibited a certain inhibitory effect on *Alternaria alternata*, with colony diameters ranging from 0.8 to 4.6 mm after 48 h, but did not completely prevent its growth. In the 30 mg/mL treatment group, no colony growth was observed after 48 h, indicating that the MFC was 30 mg/mL. This suggests that this concentration can completely kill the mycelial growth of *Alternaria alternata*.

**TABLE 1:** The effect of different concentrations of clove extract on the growth of *Alternaria alternata* after 48 h (*n* = 3).

Extract concentration (mg/mL)	Colony diameter (mm)	Inhibition rate (%)
0	7.2 ± 0.3a	0.0 ± 0.0d
15	4.6 ± 0.2b	36.1 ± 2.3c
20	2.1 ± 0.1c	70.8 ± 2.1b
25	0.8 ± 0.1d	88.9 ± 1.5a
30	0.0 ± 0.0e	100.0 ± 0.0a

Different lowercase letters following the data in the same column indicate significant differences at *P* < 0.05; Inhibition rate = (Control group colony diameter -Treatment group colony diameter)/Control group colony diameter × 100%.

#### 3.1.2 Effect on spore viability of *Alternaria alternata*

**FIGURE 1:**
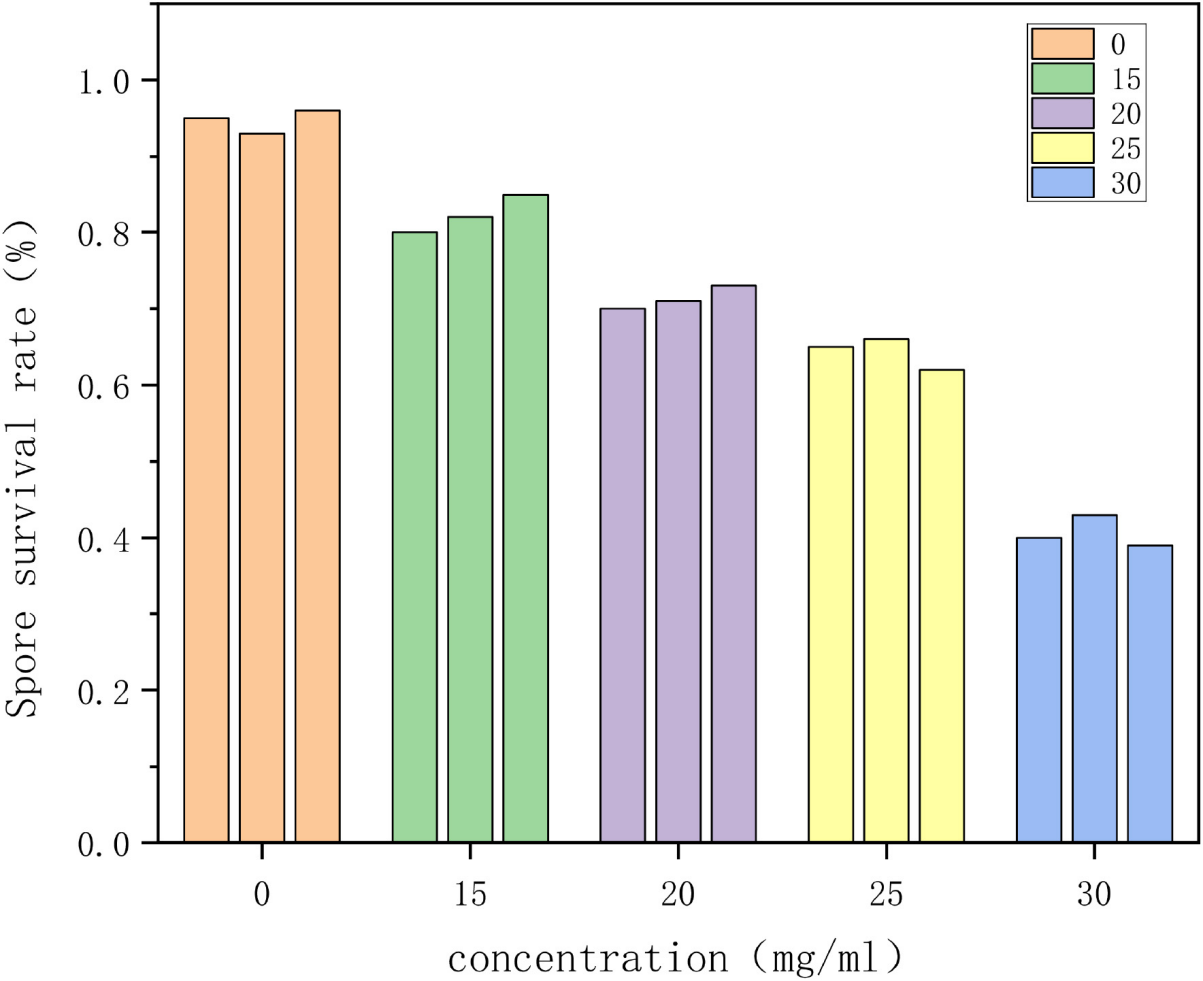
Effect of clove extract on the survival rate of *Alternaria* spores.

As shown in Figure 1, in the control group (concentration of 0), the survival rate of *Alternaria* spores reached 92.3%. Different concentrations of clove extract exhibited inhibitory effects, with the 30 mg/mL clove extract treatment group showing the most significant effect, reducing the spore survival rate to 40.0% (*P* < 0.05). MTT staining results indicated a marked decrease in the proportion of active spores in the treatment group, demonstrating that clove extract effectively reduces the reproductive capacity of *Alternaria* spores.

#### 3.1.3 Effect on membrane permeability of *Alternaria alternata*

**FIGURE 2:**
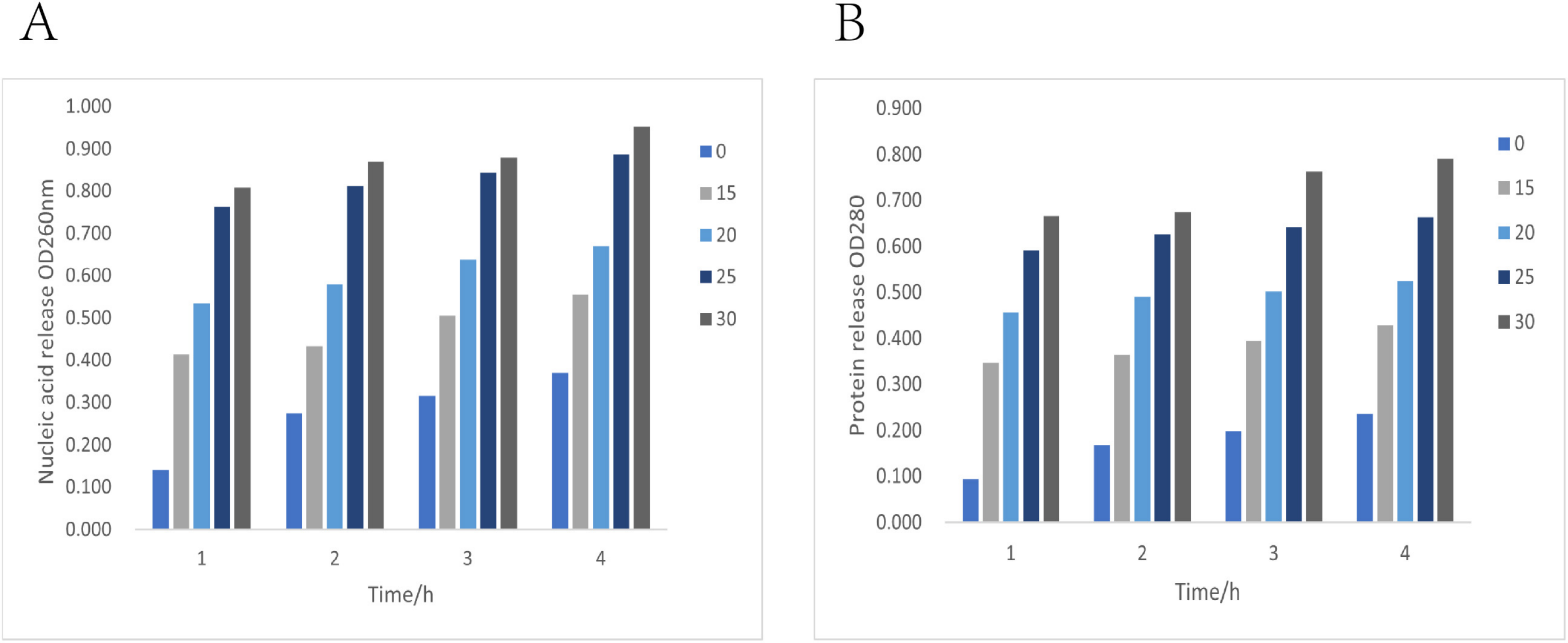
Effect of 30 mg/mL clove extract on the leakage of nucleic acids **(A)** and proteins **(B)** in *Alternaria* alternata.

The integrity of the cell membrane is the foundation of the normal physiological functions of microorganisms. The leakage of intracellular nucleic acids (OD_260_) and proteins (OD_280_) can reflect the degree of cell membrane damage (Hancock and Sahl, 2006). As shown in Figure 2, after treatment with 30 mg/mL clove extract, the leakage of fungal nucleic acid (A) and protein (B) significantly increased over time (*P* < 0.05): at 4 h, OD_260_ reached 0.952 and OD_280_ reached 0.791, which were 6.8 times and 5.2 times that of the control group, respectively. The results indicate that treatment with clove extract led to a significant increase in nucleic acid and protein leakage from the fungal cells. This observed effect suggests a disruption in cell membrane integrity or increased membrane permeability, which is associated with its antibacterial activity.

### 3.2 Changes in clove extract on the postharvest quality of the *Fragaria × ananassa* “Benihoppe” strawberries

#### 3.2.1 Hardness

**FIGURE 3:**
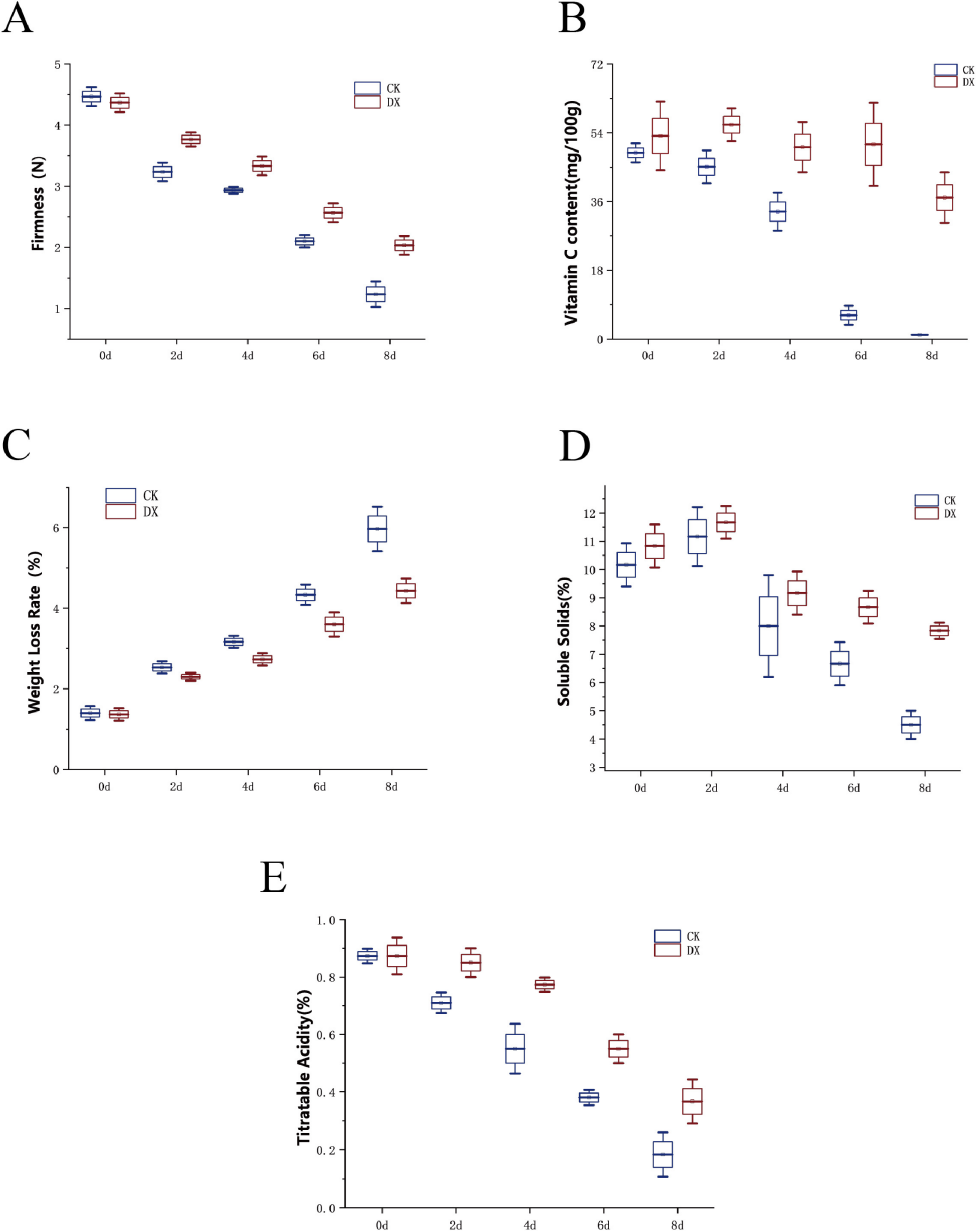
Effects of clove extract on the hardness **(A)**, VC content **(B)**, weight loss rate **(C)**, SSC **(D)**, and TA **(E)** of The Fragaria × ananassa “Benihoppe” strawberries.

Fruit firmness is a core indicator for measuring the integrity of the cellular structure of fruits (Zhang et al., 2018). The results showed that the firmness of the control group strawberry fruits decreased rapidly with storage time, dropping from an initial 4.5 to 1.2 kg/cm^2^ by the 8th day. In contrast, the firmness of the group treated with 30 mg/mL clove extract decreased at a significantly slower rate, maintaining 2.07 kg/cm^2^ on the 8th day, which was 68.3% higher than that of the control group (*P* < 0.05) (Figure 3A). This indicates that clove extract can delay the disruption of the cellular structure of strawberries.

#### 3.2.2 Vitamin C content

VC is an essential nutrient in strawberries, which is prone to degradation due to oxidation and pathogen infection (Zhang et al., 2020). In the control group, the VC content decreased from the initial 32.5 mg/100 g to 14.1 mg/100 g on the 8th day; in the treatment group, the VC content was 22.5 mg/100 g on the 8th day, which was 52.5% higher than that of the control group (*P* < 0.05) (Figure 3B). The results indicate that clove extract can reduce nutrient loss and maintain the nutritional value of strawberries.

#### 3.2.3 Weightlessness rate

The weight loss of fruits primarily stems from water evaporation and tissue decay caused by pathogen infection (Lufu et al., 2018). The results indicate that the weight loss rate of the control group reached 12.5% at day 8, while the treatment group was only 5.8%, which was 46.4% lower than the control group (*P* < 0.05) (Figure 3C). This may be related to the clove extract’s inhibition of pathogen reproduction and possibly the formation of a protective film, thereby reducing tissue decay and water evaporation.

#### 3.2.4 Soluble solids content

SSC is one of the important indicators for evaluating fruit quality. The initial increase and subsequent decrease in SSC is due to water loss and concentration in the early stage, followed by the decomposition of sugars by pathogens (Lufu et al., 2024). On the 8th day, the SSC in the CK group decreased to 8.2%, while the treatment group remained at 9.8% (*P* < 0.05) (Figure 3D), indicating that the consumption of sugars in strawberry fruits treated with clove extract was slower, further confirming its preservation effect.

#### 3.2.5 Titratable acidity content

Titratable acid, as a core component of strawberry flavor substances, directly influences the sweet and sour taste of the fruit, and is also closely related to the intensity of postharvest respiratory metabolism (Pott et al., 2020; Zhang Y. J. et al., 2021). Initially, there was no significant difference in TA content between the two groups of strawberries (*P* > 0.05), indicating consistent fruit maturity. As storage time increased, TA was consumed due to respiration, showing a continuous downward trend. At 2 days, the TA in the CK group decreased to 0.70%, while the TA in the treatment group remained stable at 0.82–0.85%. By the 8th day, the TA in the CK group was only 0.20–0.25%, and the TA in the DX group was 0.35–0.40%, significantly higher than the control group (*P* < 0.05) (Figure 3E). The data dispersion was consistently lower in the treatment group than in the control group, indicating that clove extract maintained the homeostasis of organic acids in the fruit by regulating respiration, thereby preserving flavor quality over an extended period.

### 3.3 The effect of clove extract on the microbial community on the surface of the *Fragaria × ananassa* “Benihoppe” strawberries

#### 3.3.1 Fungal community composition

**FIGURE 4:**
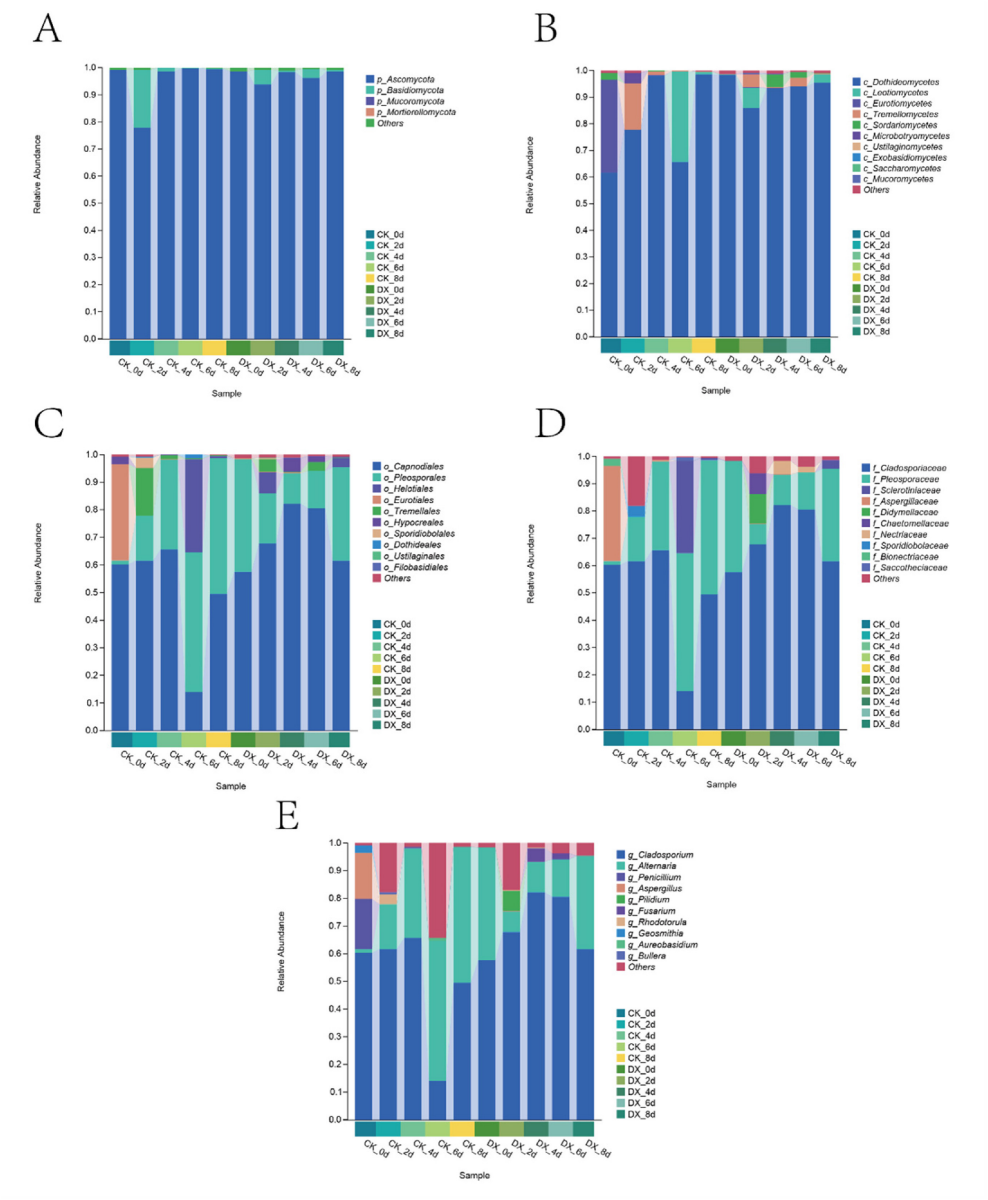
Abundance of fungi at the phylum **(A)**, class **(B)**, order **(C)**, family **(D)**, and genus **(E)** levels in stored The Fragaria × ananassa “Benihoppe” strawberries after 8 days.

At the initial stage of storage, both the CK and DX groups were dominated by Ascomycota as the absolute predominant phylum; during days 6–8, the CK group was primarily led by Ascomycota with low diversity, while the DX group exhibited a more stable community structure. Dothideomycetes was the dominant class, with significant changes observed in the CK group, whereas the DX group tended to stabilize. The changes in Cladosporium and *Alternaria*, which are pathogenic fungi, in the CK group were characterized by low initial abundance followed by a rapid increase later, whereas in the DX group, these fungi showed high initial abundance with slow growth or even a decrease later, indicating that the clove extract has a certain inhibitory effect on pathogenic fungi, especially *Alternaria*, and can maintain the stability of the fungal community (Figure 4).

### 3.3.2 Bacterial community composition

**FIGURE 5:**
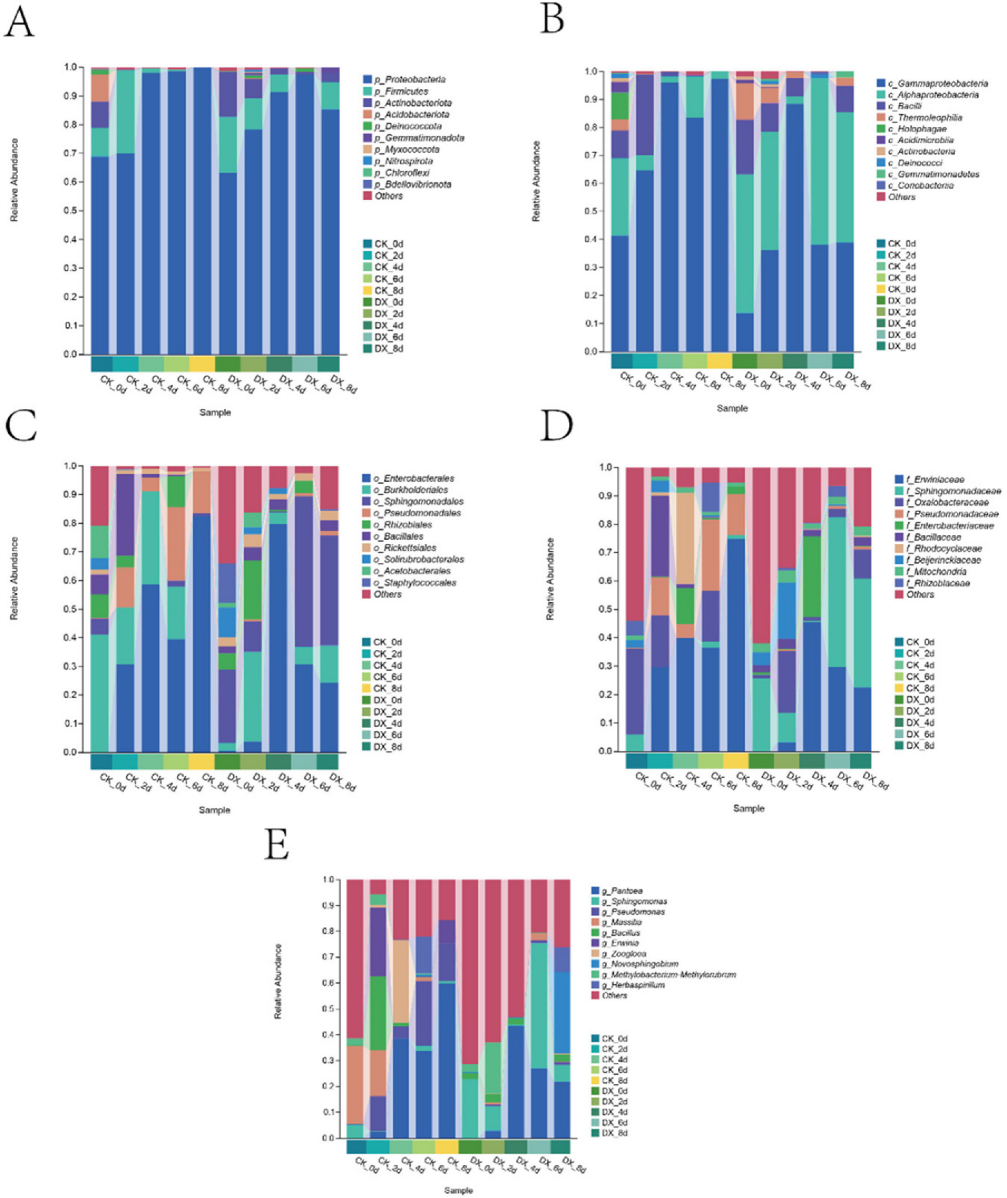
Abundance of bacterial phyla **(A)**, classes **(B)**, orders **(C)**, families **(D)**, and genera **(E)** in the Fragaria × ananassa “Benihoppe” strawberries after 8 days of storage.

In terms of bacteria (Figure 5), the abundance of spoilage bacteria (such as *Pantoea*) in the control group reached 59.97% at 8 days, while it decreased to 21.88% in the treatment group (*P* < 0.05). Meanwhile, the abundance of beneficial bacteria (such as *Bacillus*) in the treatment group (0.027%) was higher than that in the control group (0.003%). *Bacillus* can inhibit pathogenic bacteria by secreting antimicrobial substances, and the increase in its abundance may assist in enhancing the disease resistance of strawberries (Chen et al., 2018; Zhang D. et al., 2021).

#### 3.3.3 Identification of microbial community biomarkers

**FIGURE 6:**
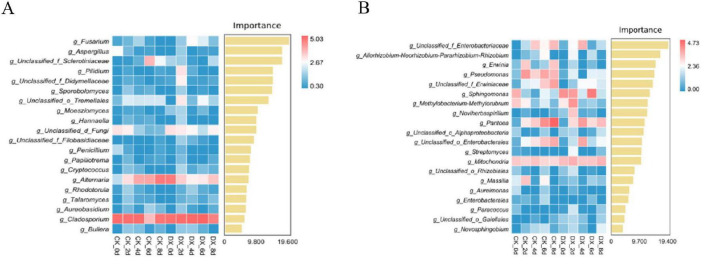
Random forest analysis of fungi **(A)** and bacteria **(B)**, with the x-axis representing species importance and the y-axis representing genus names.

Random forest analysis (Figure 6) revealed that the major pathogenic fungi *Alternaria* (importance 2.15) and bacteria *Pantoea* (importance 2.75) during plant storage are the core biomarkers distinguishing the control group from the treatment group, indicating that these genera contribute the most to the community differences during strawberry storage. Additionally, the marker characteristics of the beneficial bacteria *Bacillus* (importance 1.92) in the treatment group are prominent, further confirming its potential role in preservation (Song et al., 2023).

#### 3.3.4 The correlation between microorganisms and quality indicators (RDA analysis)

**FIGURE 7:**
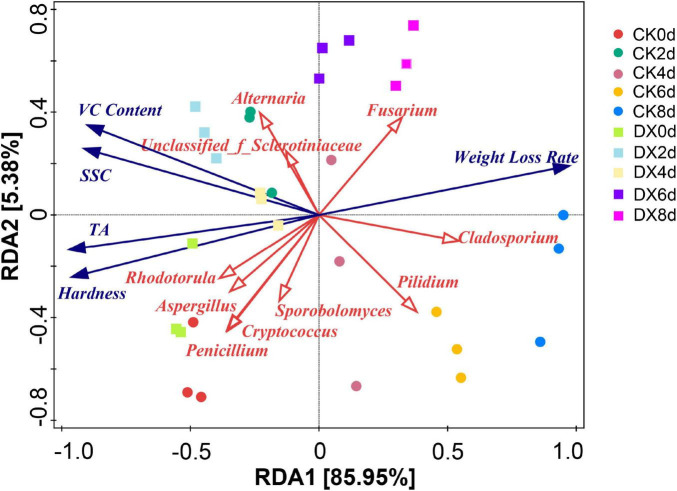
RDA ordination diagram of microbial communities and strawberry quality indicators; red arrows represent microorganisms, blue arrows represent quality indicator.

The results of Redundancy Analysis (RDA) (Figure 7) indicate that Fusarium and Cladosporium show a significant positive correlation with the Weight Loss Rate. Their proliferation accelerates water loss and dry matter consumption in strawberries, directly promoting fruit weight loss and deterioration. Quality indicators such as VC Content, SSC, TA, and Hardness are negatively correlated with fungi like *Alternaria* and Unclassified_f_Sclerotiniaceae. The enrichment of these fungi exacerbates nutritional degradation and the deterioration of taste and texture in strawberries (Zhang N. et al., 2021). In the initial stages, the sample points of DX0d and DX2d were closer to the direction of high-quality indicators such as VC Content, SSC, TA, and Hardness, and farther away from pathogenic fungi like *Alternaria*. This may indicate that clove extract can inhibit the colonization of spoilage fungi, maintaining the nutritional and quality homeostasis of strawberries. In the later stages, the sample points of CK4d-8d gradually clustered toward spoilage fungi such as Fusarium and Cladosporium, as well as the high weight loss rate area, while the sample points of DX4d-8d overall deviated from this trend and were closer to potentially beneficial fungi like Rhodotorula and Cryptococcus. It is indicated that the clove extract delayed the synergistic process of “proliferation of spoilage fungi—quality deterioration” by regulating community succession (Ayoubi et al., 2017).

## 4 Discussion

### 4.1 The antibacterial mode of action and application potential of clove extract against *Alternaria* alternata on strawberries from antifungal experiments to the verification of actual preservation effects

#### 4.1.1 The material basis and action targets of antibacterial activity

The following discussion interprets the observed patterns from our experiments. We correlate our findings with the existing literature to suggest possible explanations for the preservative effect, while acknowledging that causal relationships require further validation. This study clearly determined that the minimum fungicidal concentration (MFC) of the clove extract against the *Fusarium* spore is 30 mg/mL. Its antifungal activity is likely closely related to the phenolic and aldehyde-ketone compounds abundant in clove. These plant-derived active components are the core substances for the antibacterial effect of essential oils and can interfere with the metabolism of pathogenic bacteria through multiple pathways (Burt, 2004). The antifungal activity of clove extract is likely attributed to its complex mixture of bioactive compounds, such as eugenol. The observed reduction in spore viability and increase in cell membrane permeability (nucleic acid/protein leakage) in our study align with a mode of action that involves interference with microbial cell integrity and physiology. Compared to single-target chemical fungicides, the multi-component nature of plant extracts like clove may exert inhibitory effects through several concurrent pathways, potentially reducing the risk of resistance development.

#### 4.1.2 From the antifungal experiments to the verification of the actual preservation effect

When the clove extract was applied to preserve strawberries, the treated group showed significantly fewer obvious disease spots throughout the process, verifying its efficacy in controlling *Alternaria alternata* in practical scenarios. This study revealed the potential value of clove extract in replacing chemical fungicides for post-harvest strawberries, and its application in pre-cooling, coating and other processes to achieve the goal of no chemical residues.

### 4.2 The regulatory effect of clove extract on the microbial community of strawberry spoilage

#### 4.2.1 The mode of actions of community structure reshaping and preservation

The results of high-throughput sequencing and redundancy analysis (RDA) indicated that the clove extract significantly altered the microbial community succession on the surface of strawberries. In the middle and late stages of storage, the abundance of bacterial genera strongly associated with spoilage in the control group rapidly increased, while the growth of such spoilage bacteria in the treatment group could be effectively inhibited and the beneficial bacterial genera maintained a relatively stable abundance in the treatment group.

This mode of action can be summarized as: The clove extract achieves micro-ecological balance by inhibiting the growth and reproduction of spoilage bacteria and enriching beneficial bacteria. Beneficial bacteria can secrete extracellular polysaccharides, antimicrobial peptides and other metabolites, which further inhibit the growth of pathogenic bacteria. The synergistic effect of the extract and beneficial bacteria achieves the preservation effect, providing a theoretical basis for the subsequent development of natural preservatives + probiotic compound technology.

#### 4.2.2 The coordinated response of microbial communities to quality indicators

The RDA analysis visually presents the coupling relationship between the microbial community and the quality indicators: the spoilage bacteria such as *Fusarium* and *Cladosporium* have a significantly positive correlation with the weight loss rate (the explained degree of the RDA1 axis is 85.95%); their proliferation accelerates the loss of fruit moisture (the weight loss rate of CK8d reached 6.0%, and that of the DX group was 4.4%); while the quality indicators such as VC content and hardness have a negative correlation with the spoilage bacteria such as *Alternaria* and a positive correlation with the beneficial bacteria such as Rhodotorula. The correlation observed in RDA suggests a link between the shifts in microbial community structure induced by clove extract treatment and the better maintenance of fruit quality indicators. By inhibiting the spoilage bacteria, reducing their degradation of fruit nutrients (such as VC and titratable acid) (the retention rate of VC content in the DX group at 8 days was 37%, while that in the CK group was 1.1%), delaying the activity of cell wall degrading enzymes, and maintaining the hardness of the fruit (the retention rate of hardness in the DX group at 8 days was 2.0%, while that in the CK group was 1.2%). This synergistic response mode of action provides a thought-provoking approach for the research and development of fruit and vegetable preservation technology.

### 4.3 The expansion value of the research system and the industrial application prospects

#### 4.3.1 The extension and improvement of the technical system

This study has established a technical chain for *in vitro* preservation. It can further integrate technologies such as GC-MS and LC-MS to identify the core antibacterial components in the clove extract, analyze the target effect relationship of these components on different spoilage bacteria, develop targeted antibacterial precision preservatives, or combine the clove extract with chitosan coating, gas-improved packaging, and construct a composite preservation system of biological preservatives + physical treatment + gas environment. This study can also explore the variety-specific preservation effects of clove extract for main cultivated strawberry varieties such as “Hongyan” and “Zhangji,” clarify the key influencing factors, and achieve precise preservation.

#### 4.3.2 The contribution to the fruit and vegetable disease prevention and control system

This research has the characteristics of universality and systematic application. *Cladosporium* is a common pathogen of fruit fruits. The broad-spectrum antibacterial property of the clove extract can be extended to the prevention and control of multiple types of fruit and vegetable complex diseases. As a regulator of fruit communities, the clove extract provides a new ecological control idea for post-harvest disease prevention and control of fruits and vegetables. In the future, an integrated system of “natural extracts + microbialomics monitoring + intelligent preservation warning” can be constructed to achieve precise and green post-harvest disease prevention and control of fruits and vegetables. Therefore, the innovative value of this research is not limited to strawberries, but can also provide a reference for the post-harvest disease prevention and control of other berry-type fruits (such as blueberries and raspberries).

## 5 Conclusion and outlook

This study demonstrates that treatment with clove extract delayed the postharvest deterioration of “Benihoppe” strawberries. This effect was correlated with (i) direct inhibition of the target fungus *A. alternata*, (ii) a shift in the fruit surface microbial community characterized by reduced relative abundance of certain spoilage organisms and increased abundance of potential beneficial bacteria, and (iii) better retention of key fruit quality parameters (firmness, VC, weight, etc.). This result provides a natural and efficient technical solution for post-harvest preservation of strawberries, and also reveals the mode of action by which natural extracts regulate the post-harvest microbiome of fruits and vegetables from the perspective of microbial genetics. Based on the results of this study, future research can focus on analyzing the mode of action between the clove extract and the surface microorganisms of strawberry fruits, clarifying the molecular mode of action of bacterial regulation, and subsequently developing microcapsulated and nanized preparations of the clove extract to enhance its stability and duration. Eventually, industrialization can be carried out to test the potential of the technology’s large-scale application. Achieving a complete process from preliminary basic research to final industrialization, this study is expected to provide key support for the green control of post-harvest diseases in fruits and vegetables, and promote the comprehensive upgrade of the natural preservation technology system (Guo et al., 2020; Jin et al., 2018; Chernetsova and Morlock, 2011).

## Data Availability

The data are available from the corresponding author upon reasonable request.
